# STING Ligand-Mediated Priming of Functional CD8^+^ T Cells Specific for HIV-1-Protective Epitopes from Naive T Cells

**DOI:** 10.1128/JVI.00699-21

**Published:** 2021-07-26

**Authors:** Nozomi Kuse, Tomohiro Akahoshi, Masafumi Takiguchi

**Affiliations:** a Tokyo Joint Laboratory and Division of International Collaboration Research, Joint Research Center for Human Retrovirus Infection, Kumamoto University, Kumamoto, Japan; b Center for AIDS Research, Kumamoto University, Kumamoto, Japan; Emory University

**Keywords:** CD8^+^ T cells, HIV-1, HLA, STING ligand, conserved regions, naive T cells, protective epitope

## Abstract

Functional HIV-1-specific CD8^+^ T cells primed from naive T cells are expected to act as effector T cells in a “shock-and-kill” therapeutic strategy for an HIV-1 cure since less functional HIV-1-specific CD8^+^ T cells are elicited from memory T cells in HIV-1-infected individuals on combined antiretroviral therapy (cART). CD8^+^ T cells specific for HIV-1 conserved and protective epitopes are candidates for such T cells. We investigated the priming with STING ligand of CD8^+^ T cells specific for HLA-B*52:01 or HLA-C*12:02-restricted protective epitopes from naive T cells. STING ligand 3′3′-cGAMP effectively primed CD8^+^ T cells specific for 3 of 4 HLA-B*52:01-restricted epitopes but failed to prime those specific for all 3 HLA-C*12:02-restricted epitopes from the naive T cells of HIV-1-uninfected individuals having an HLA-B*52:01-C*12:02 protective haplotype. These HLA-B*52:01-restricted CD8^+^ T cells had a strong ability to suppress HIV-1 replication and expressed a high level of cytolytic effector molecules. The viral suppression ability of these T cells was significantly correlated with the expression level of perforin and showed a trend for a positive correlation with the expression level of CD107a. The present study highlighted the priming with STING ligand of functional CD8^+^ T cells specific for protective epitopes, which T cells would contribute as effector T cells to a shock-and-kill therapy.

**IMPORTANCE** The current “shock-and-kill” therapeutic strategy for HIV cure has been directed toward eliminating latent viral reservoirs by reactivation of latent reservoirs with latency-reversing agents followed by eradication of these cells by immune-mediated responses. Although HIV-1-specific T cells are expected to eradicate viral reservoirs, the function of these T cells is reduced in HIV-1-infected individuals with long-term cART. Therefore, priming of HIV-1-specific T cells with high function from naive T cells is to be expected in these individuals. In this study, we demonstrated the priming with STING ligand 3′3′-cGAMP of CD8^+^ T cells specific for HIV-1-protective epitopes from naive T cells. cGAMP primed CD8^+^ T cells specific for 3 HLA-B*52:01-restricted protective epitopes, which cells expressed a high level of cytolytic effector molecules and effectively suppressed HIV-1 replication. The present study suggested that the priming with STING ligand of functional CD8^+^ T cells specific for protective epitopes would be useful in a therapy for an HIV-1 cure.

## INTRODUCTION

Despite successful suppression of HIV-1 replication by optimal combined antiretroviral therapy (cART), cART cannot completely eradicate HIV-1 due to the persistence of latently infected cells harboring replication-competent proviruses in individuals on treatment (1–4). The current therapeutic strategy has been directed toward eliminating latent viral reservoirs via a shock-and-kill approach, which is based on reactivation of latent reservoirs (the shock) with latency-reversing agents followed by eradication of these cells (the kill) by immune-mediated responses (5–7). Recent studies suggested that HIV-1-specific T cells also play a key role in purging viral reservoirs in HIV-1-infected individuals on cART (8, 9). However, the number and function of HIV-1-specific effector and memory T cells are lost and/or reduced in HIV-1-infected individuals receiving long-term cART (10–17). Therefore, the induction of *de novo* CD8^+^ T cells from naive T cells would be one option for eradicating latently HIV-1-infected cells, while functional recovery of HIV-1-specific effector and memory T cells may be another one.

Previous phase III clinical trials of T-cell vaccines showed no protective effect against an HIV-1 infection, even though the vaccine induced HIV-1-specific T-cell responses (18–20), indicating that the CD8^+^ T cells induced by the vaccines had insufficient ability to suppress HIV-1 replication. HIV-1-specific CD8^+^ T cells generated during a chronic infection also showed defects in antiviral function and skewed differentiation of HIV-1-specific T cells (21, 22). These studies emphasized the requirement for the induction of CD8^+^ T cells with high functional properties for both an AIDS vaccine and a functional cure. A previous study demonstrated that agonists for the innate sensor STING (stimulator of interferon [IFN] genes) such as cyclic dinucleotides (cGAMP) more effectively enhanced the induction of effector CD8^+^ T cells specific for a Melan-A epitope from naive T cells derived from healthy individuals and induced viral antigen-specific T cells in mice immunized with viral antigens (23). On the other hand, our recent study on priming functional HIV-1-specific CD8^+^ T cells from naive T cells showed that 3′3′-cGAMP could prime highly functional NefRF10-specific CD8^+^ T cells from naive T cells derived from healthy individuals, whereas LPS effectively primed NefRF10-specific CD8^+^ T cells having a weak function (24).

The accumulation of immune escape mutations in circulating HIV-1 is one of the hurdles for AIDS vaccine development and cure treatment using T-cell immunity (25). CD8^+^ T cells specific for several conserved Gag and Pol regions have a strong ability to suppress HIV-1 replication both *in vivo* and *in vitro* (26–30). The responders to these epitopes showed significantly higher CD4 counts and lower plasma viral loads than nonresponders, suggesting that T cells specific for these epitopes play a protective role against HIV-1 infection *in vivo* (26, 28–30). It is therefore expected that CD8^+^ T cells specific for these protective epitopes may have the ability to eradicate HIV-1-infected cells if they are primed with cGAMP from naive T cells. Our previous studies showed that the HLA-B*52:01-C*12:02 haplotype, which is the most prevalent haplotype in Japanese individuals (approximately 20%), is strongly associated with a good clinical outcome (31) and demonstrated that 4 HLA-B*52:01-restricted (GagRI8, GagMI8, GagWV8, and PolSI8) and 2 HLA-C*12:02-restricted (PolIY11 and NefMY9) protective and immunodominant cytotoxic T lymphocyte (CTL) epitopes effectively suppress HIV-1 replication both *in vitro* and *in vivo* (26, 27, 32–34). T cells specific for these protective epitopes may have the ability to eliminate the latent viral reservoir in HIV-1-infected Japanese individuals carrying the HLA-B*52:01-C*12:02 haplotype on cART if the reservoir virus is activated.

Our previous study showed priming of highly functional T cells specific for only one HIV-1 epitope, NefRF10 (24). Therefore, it still remains unknown whether cGAMP can prime functional CD8^+^ T cells specific for other HIV-1 epitopes from naive T cells. Since NefRF10 is not a protective epitope, in the present study, we sought to prime CD8^+^ T cells specific for HLA-B*52:01-restricted and HLA-C*12:02-restricted protective epitopes from HIV-1-seronegative individuals carrying the HLA-B*52:01-C*12:02 haplotype by using the STING ligand cGAMP and analyzed the function of these T cells. These HIV-1-specific T cells would be candidates of effector T cells to eradicate the latent reservoirs in a “shock-and-kill” therapy.

## RESULTS

### Effect of STING ligand on priming of CD8^+^ T cells specific for HLA-B*52:01-restricted HIV-1-protective epitopes from naive T cells.

**FIG 1:**
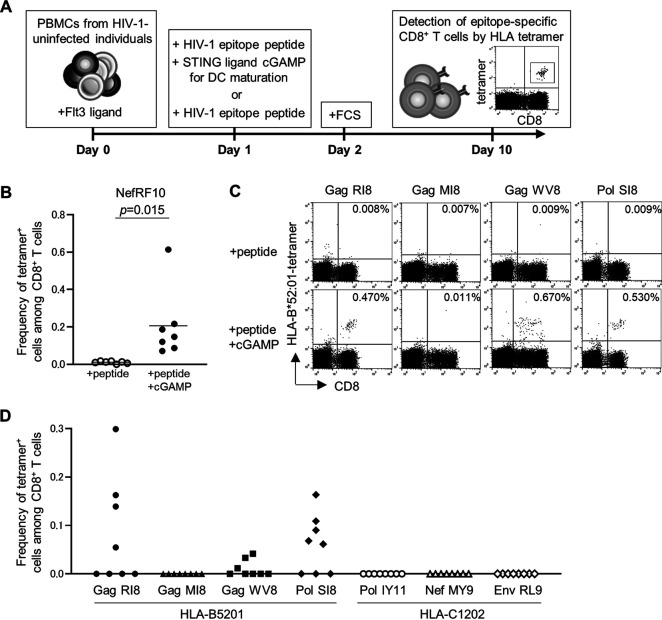
Priming of CD8^+^ T cells specific for protective epitopes from HIV-1-seronegative individuals having the HLA-B*52:01-C*12:02 haplotype. CD8^+^ T cells specific for HLA-A*24:02-restricted NefRF10 and those specific for 4 HLA-B*52:01-restricted epitopes (GagRI8, GagWV8, GagMI8, and PolSI8) and 3 HLA-C*12:02-restricted ones (PolIY11, NefMY9, and EnvRL9) from 7 HIV-1-seronegative individuals having HLA-A*24:02 and 8 ones having the HLA-B*52:01-C*12:02 haplotype, respectively, were primed with 3′3′-cGAMP. (A) Schematic representation of *in vitro* priming of epitope-specific CD8^+^ T cells from naive T cells by using 3′3′-cGAMP. Epitope-specific T cells were primed from each individual in triplicate. (B) The frequency of HLA-A*24:02-restricted NefRF10-specific T cells from 7 HIV-1-seronegative HLA-A*24:02^+^ individuals. Each dot represents the average (*n* = 3) frequency of tetramer^+^ cells among the CD8^+^ T cells in each individual. Horizontal bars indicate median values. Statistical analysis was conducted by use of the paired *t* test. (C) Representative results of tetramer staining of HLA-B*52:01-restricted epitope-specific CD8^+^ T cells primed from donors U-70 (GagRI8), U-62 (PolSI8), and U-30 (GagMI8 and GagWV8). The frequency of the tetramer^+^ cells among the CD8^+^ T-cell population is indicated. (D) Summarized results of the frequency of 3′3′-cGAMP-induced epitope-specific CD8^+^ T cells from the 8 individuals having the HLA-B*52:01-C*12:02 haplotype. GagRI8-specific and PolSI8-specific T cells were not induced by the peptide in 3 individuals in whom the specific T cells were primed by both 3′3′-cGAMP and the peptide. Each dot represents the average (*n* = 3) frequency of tetramer^+^ cells among the CD8^+^ T cells in each individual.

Our previous study demonstrated effective priming of NefRF10-specifc HLA-A*24:02-restricted T cells from naive T cells by 3′3′-cGAMP together with immunodominant NefRF10 epitope peptide (24). However, in that study, we did not show that NefRF10-specific T cells were induced by only NefRF10 peptide. We therefore presently investigated the priming of NefRF10-specific T cells from naive T cells not only by 3′3′-cGAMP and NefRF10 peptide but also by only NefRF10 peptide. Peripheral blood mononuclear cells (PBMCs) from 7 HIV-1-seronegative HLA-A*24:02^+^ individuals were stimulated with Flt3 ligand for 24 h, followed by stimulation with 3′3′-cGAMP and NefRF10 peptide or with NefRF10 peptide only. The induction of NefRF10-specific T cells was assessed by a flow cytometric analysis using HLA-A*24:02-NefRF10 tetramers after a 10-day culture period (Fig. 1A). Specific T cells were found in culture cells stimulated with 3′3′-cGAMP and NefRF10 peptide from all of the individuals (mean frequency of NefRF10-specific T cells ± 2 standard deviations [SD], 0.206% ± 0.186%), whereas they were not detected in those stimulated with only RF10 peptide (mean frequency of NefRF10-specific T cells ± 2 SD, 0.011% ± 0.006% [Fig. 1B]).

Though NefRF10 is known to be an immunodominant epitope (35), it is unknown if STING ligand 3′3′-cGAMP can prime T cells specific for other HIV-1 immunodominant epitopes from naive T cells. We first analyzed 4 HLA-B*52:01-restricted immunodominant epitopes (GagRI8, GagMI8, GagWV8, and PolSI8). To investigate the ability of 3′3′-cGAMP to prime CD8^+^ T cells specific for these immunodominant epitopes from naive T cells, we recruited 8 HIV-1-seronegative individuals having the HLA-B*52:01-C*12:02 haplotype. On day 10 after the start of cultures, we measured epitope-specific CD8^+^ T cells by staining the cultured cells with HLA-B*52:01-peptide tetramers. Representative results are shown in Fig. 1C. CD8^+^ T cells specific for GagRI8, GagWV8, and PolSI8 were successfully primed with 3′3′-cGAMP from 50% (4/8), 25% (2/8), and 62% (5/8) of HIV-1-seronegative individuals, respectively (Fig. 1D), whereas those specific for GagMI8 were not primed with this ligand from any of the individuals (Fig. 1D).

**FIG 2:**
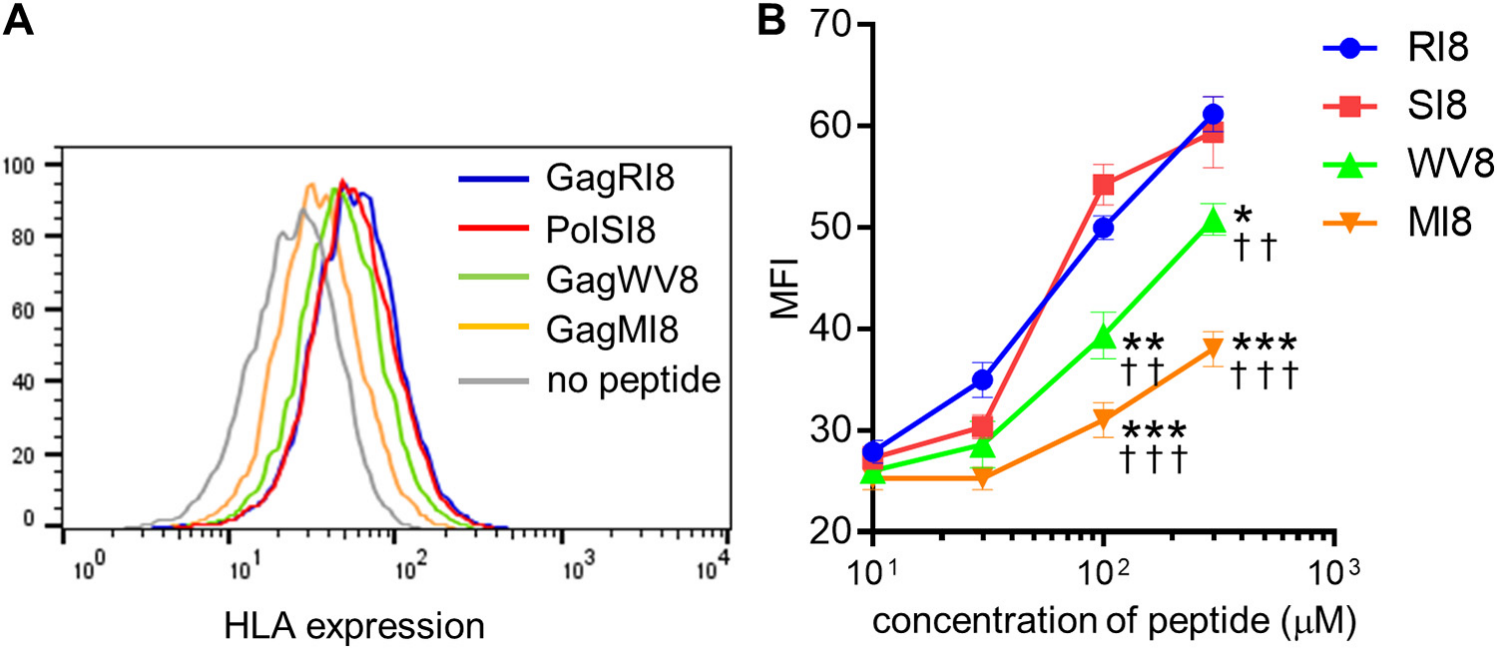
Ability of epitope peptides to bind to HLA-B*52:01 molecules. The ability of 4 peptides (GagRI8, GagMI8, GagWV8, and PolSI8) to bind to HLA-B*52:01 was measured by performing the HLA class I stabilization assay using RMA-S-B*52:01 cells. (A) Representative staining data on HLA-B*52:01 expression at a 300 μM concentration of each epitope peptide. (B) Ability of peptides at concentrations from 0 μM to 300 μM to bind to HLA-B*52:01. Data are shown as the means and SD of triplicate assays. The MFI of RMA-S-B*52:01 cells without peptide (0 μM) was 25.3 ± 0.2. Statistical analysis was performed by using the unpaired *t* test. Asterisks and daggers indicate significant difference (*, *P* < 0.05; **, *P < *0.01; ****, *P* < 0.001 [SI8 versus WV8 or MI8]; ††, *P < *0.01; †††, *P < *0.001 [RI8 versus WV8 or MI8]).

We next investigated the ability of these epitope peptides to bind to HLA-B*52:01 by performing an HLA stabilization assay using RMA-S-HLA-B*52:01 cells (Fig. 2A). GagRI8 and PolSI8 peptides bound to HLA-B*52:01 with similar binding affinities, whereas the affinity of the GagWV8 peptide for HLA-B*52:01 was significantly weaker than that of GagRI8 and PolSI8 (Fig. 2B). In contrast, the affinity of the GagMI8 peptide for HLA-B*52:01 was much weaker than that of the other 3 epitopes (Fig. 2B). Thus, the binding affinity of these epitope peptides was correlated with the efficacy of specific CD8^+^ T-cell induction from naive T cells in this priming system.

### Failure of STING ligand-mediated priming of CD8^+^ T cells specific for HLA-C*12:02-restricted HIV-1 epitopes.

We further investigated the ability of the STING ligand 3′3′-cGAMP to prime CD8^+^ T cells specific for HLA-C*12:02-restricted ones from naive T cells derived from the same individuals (indicated above) who had been analyzed for the priming of HLA-B*52:01-restricted CD8^+^ T cells. In addition to 2 protective and immunodominant epitopes (PolIY11, NefMY9), HLA-C*12:02-restricted CTL immunodominant epitope EnvRL9 (36) was also used in this study. Specific CD8^+^ T cells for these epitopes were analyzed by using HLA-C*12:02-tetramers for each epitope. CD8^+^ T cells specific for these 3 epitopes were not primed from any of the 8 HIV-seronegative individuals having the HLA-B*52:01-C*12:02 haplotype (Fig. 1D).

**FIG 3:**
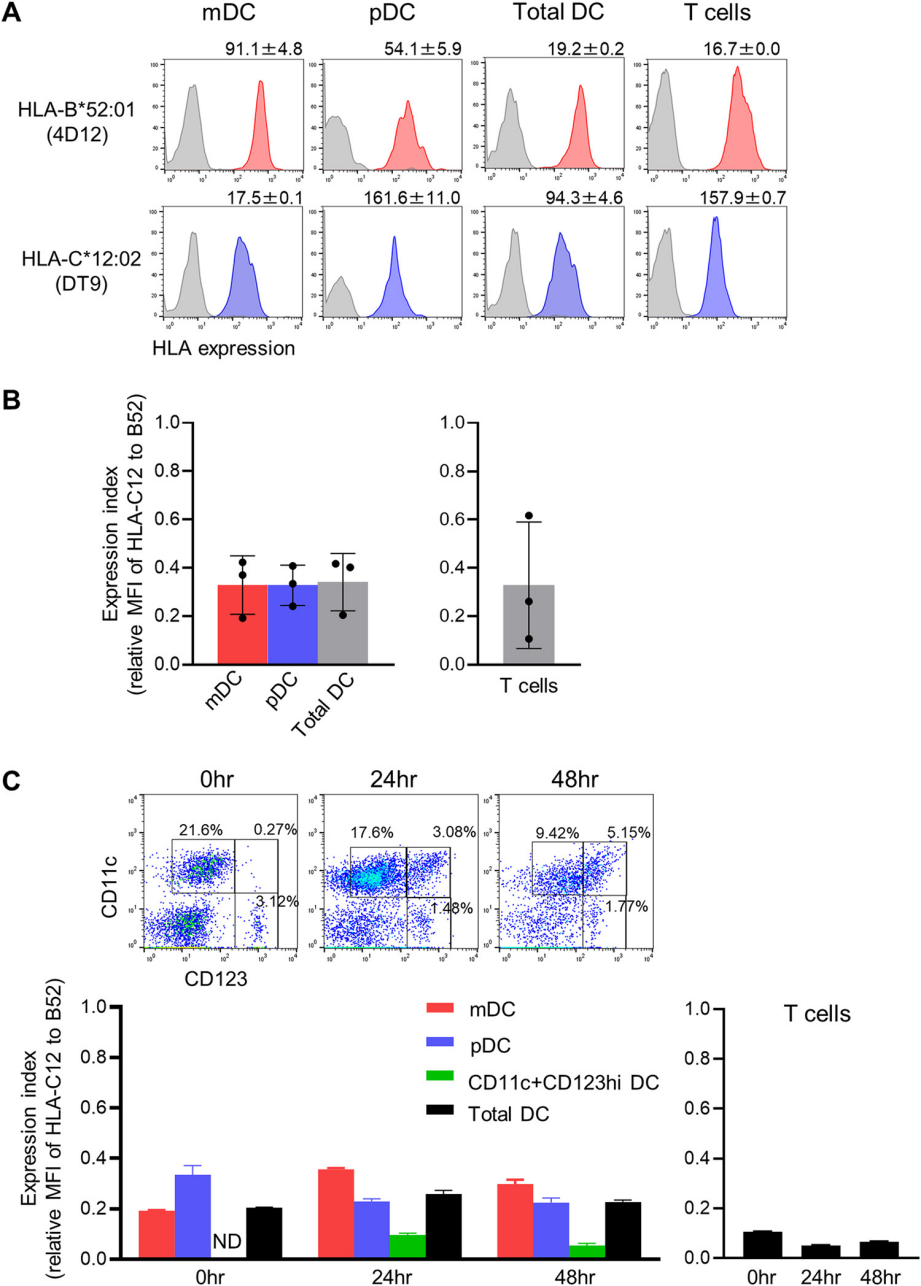
Comparison of cell surface expression between HLA-B*52:01 and HLA-C*12:02 molecules on DCs and T cells from the same individual. PBMCs from 3 individuals who were homozygous for the HLA-B*52:01-C*12:02 haplotype were stimulated with Flt3 ligand for 24 h (0 h). (A) Representative staining data on HLA-B*52:01 and HLA-C*12:02 expression on DCs and T cells at 0 h. After the gating of mDCs (HLA-DR^+^ Lin^−^ CD11c^+^ CD123^lo^ cells), pDCs (HLA-DR^+^ Lin^−^ CD11c^−^ CD123^hi^ cells), and T cells (CD3^+^ cells), the expression levels of HLA-B*52:01 and HLA-C*12:02 were analyzed by staining with anti-B5 MAb 4D12 (red), anti-HLA-C MAb DT9 (blue), and isotype control (gray) and were compared in terms of relative mean fluorescence intensity (MFI). (B) Expression levels at 0 h of HLA-B*52:01 and HLA-C*12:02 on mDCs, pDCs, total DCs (mDCs, pDCs, and CD11c^+^ CD123^hi^ DCs), and T cells from 3 individuals who were homozygous for the HLA-B*52:01-C*12:02 haplotype were compared in terms of expression index. The expression index was calculated as follows: relative MFI of HLA-C*12:02/relative MFI of HLA-B*52:01. (C) Time course of HLA expression index after stimulation with 3′3′-cGAMP. PBMCs from an individual who was homozygous for the HLA-B*52:01-C*12:02 haplotype were stimulated with Flt3 ligand for 24 h (0 h), followed by stimulation with 3′3′-cGAMP for 24 h (24 h) or 48 h (48 h). Representative staining data on DCs for each time is shown in the upper portion. ND, no data. Concentrations of 4D12 and DT9 MAbs were determined as shown in Table 1. The relative MFI was calculated as the MFI of cells stained with HLA antibody/MFI of cells stained with the isotype control. Experiments were performed in triplicate.

Since it is well known that the expression level of HLA-C molecules on the cell surface is lower than that of HLA-A and -B ones (37–39), we speculated that the low expression level of HLA-C*12:02 molecules on the antigen-presenting cells resulted in failure of priming of CD8^+^ T cells specific for HLA-C*12:02-restricted epitopes from naive T cells. So, we investigated the expression levels of HLA-C*12:02 and HLA-B*52:01 on dendritic cells (DCs). First, to quantify the relative strength of binding of anti-B5 (HLA-B*51 and -B*52) monoclonal antibody (MAb) (4D12) and anti-HLA-C MAb (DT9), we determined the concentration of these MAbs that provided the same affinity by using TP25.99 anti-HLA class I α3 domain MAb (Table 1). We then analyzed the expression levels of HLA-B*52:01 and HLA-C*12:02 on plasmacytoid DCs (pDCs), myeloid DCs (mDCs), and T cells from 3 HIV-1-seronegative individuals homozygous for the HLA-B*52:01-HLA-C*12:02 haplotype by using these 4D12 and DT9 MAbs (Fig. 3A). The expression of HLA-C*12:02 on both DCs and T cells was approximately 3 to 5 times lower than that of HLA-B*52:01 on those cells (Fig. 3B). We further analyzed the expression levels of HLA-B*52:01 and HLA-C*12:02 on DCs and T cells 24 h and 48 h after stimulation with 3′3′-cGAMP. The expression of HLA-C*12:02 on both DCs and T cells was approximately 3 to 10 times lower than that of HLA-B*52:01 on those cells (Fig. 3C). These results taken together suggest that a low expression level of HLA-C*12:02 molecules may be one of the important reasons why CD8^+^ T cells specific for HLA-C*12:02-restricted epitopes were not primed from naive T cells.

**TABLE 1:** Normalized binding ability of 4D12 MAb toward HLA-B*52:01 and that of DT9 MAb toward HLA-C*12:02^*a*^

Antibody	MFI		Relative MFI		Relative binding ratio	
	B*52:01	C*12:02	B*52:01	C*12:02	B*52:01	C*12:02
4D12	189.4	8.5	61.8	2.0	1.2	0.0
DT9	3.7	155.3	1.2	37.2	0.0	1.2
TP25.99	153.6	120.1	50.2	28.8	1.0	1.0
Negative	3.0	4.1	1.0	1.0	—	—

a The relative mean fluorescence intensities (MFIs) of 721.221-HLA-B*52:01 cells stained with 4-times-diluted hybridoma culture medium containing 4D12 MAb or 307,200-times-diluted ascites fluid containing TP25.99 MAb were 61.8 ± 1.1 and 50.1 ± 7.8, respectively; whereas the relative MFIs of 721.221-HLA-C*12:02 cells stained with 192-times-diluted culture medium of DT9 MAb or 307,200-times-diluted ascites fluid containing TP25.99 were 37.2 ± 0.5 and 28.8 ± 0.0, respectively. These concentrations of antibodies provided the same relative binding ratio of 1.2. The concentration of 4D12 or DT9 MAb with a 1.2 relative binding ratio was selected for further analysis in terms of comparison of cell surface expression between HLA-B*52:01 and HLA-C*12:02 molecules in Fig. 3. “Negative” means that cells were not stained with MAbs.

### Ability of 3′3′-cGAMP-primed CD8^+^ T cells specific for protective epitopes to suppress HIV-1 replication.

**FIG 4:**
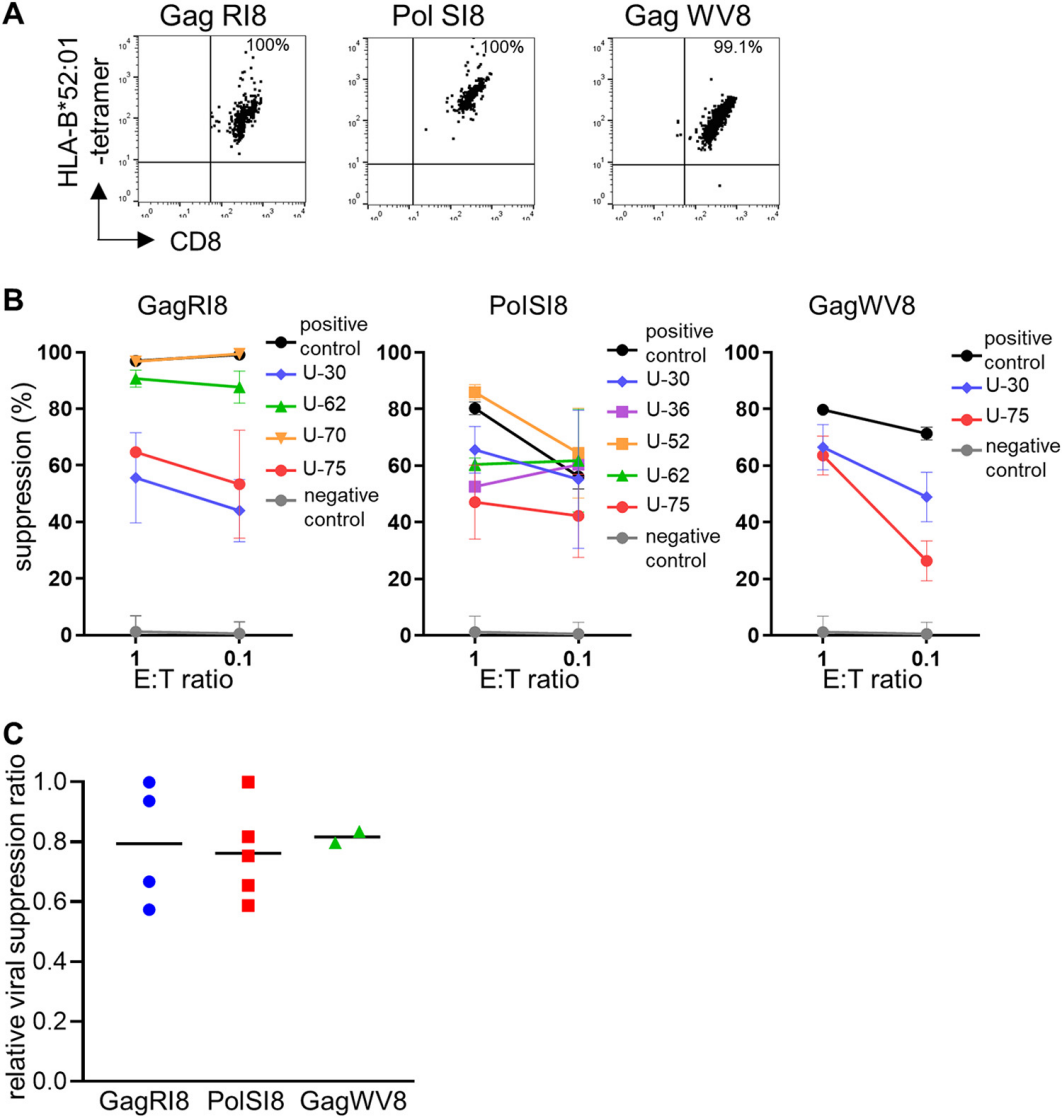
Viral suppression ability of HLA-B*52:01-restricted protective epitope-specific CD8^+^ T cells primed with 3′3′-cGAMP from naive T cells. (A) Representative results of tetramer staining of HLA-B*52:01-restricted CD8^+^ T-cell lines specific for GagRI8, GagWV8, or PolSI8 established from an HIV-1-seronegative individual (U-30). The T-cell lines were stained with HLA-B*52:01 tetramer specific for each epitope at a concentration of 100 nM. (B and C) Ability of 3′3′-cGAMP-primed T-cell lines specific for GagRI8, GagWV8, and PolSI8 to suppress the replication of HIV-1. NL4-3-infected CD4^+^ T cells from an HLA-B*52:01^+^ donor were cocultured at different E:T ratios with 3′3′-cGAMP-primed T-cell lines or with the control CTL clones, 12B (GagRI8 specific), D3 (PolSI8 specific), C5 (GagWV8 specific), and 113 (HLA-B*35:01-restricted Env77-85), established from HIV-1-infected individuals. Percent inhibition of 3′3′-cGAMP-primed T-cell lines and control clones is indicated (B). GagRI8-specific, PolSI8-specific, or GagWV8-specific T-cell clone and HLA-B*35:01-restricted Env77-85 derived from HIV-1-infected individuals were used as positive and negative controls, respectively. Data are shown as the means and SD of triplicate assays. (C) Evaluation of viral suppression ability of CD8^+^ T-cell lines primed with 3′3′-cGAMP in terms of relative viral suppression ratio. The relative viral suppression ratio was calculated as follows: percent inhibition of 3′3′-cGAMP-primed T-cell line/that of positive control CTL clone established from HIV-1-infected individuals. The results at the E:T ratio of 1:1 are shown. Horizontal bars indicate median values.

Since the frequency of GagRI8-, GagWV8-, and PolSI8-specific CD8^+^ T cells among total primed T cells was too low to analyze the function of the primed T cells (Fig. 1), we established CD8^+^ T-cell lines specific for these epitopes for further functional analysis. Epitope-tetramer^+^ CD8^+^ T cells were sorted and then cultured for 2 additional weeks. GagRI8-, GagWV8-, and PolSI8-specific CD8^+^ T-cell lines were successfully established from 4, 2, and 5 HIV-1-seronegative individuals, respectively, having the HLA-B*52:01-C*12:02 haplotype (Fig. 4A). We investigated the ability of these primed CD8^+^ T-cell lines to suppress the replication of HIV-1, which suppression is an indicator of the efficacy of CD8^+^ T cells to control HIV-1. The results of the viral suppression assay showed that primed T-cell lines specific for all of these epitopes effectively suppressed HIV-1 replication in primary CD4^+^ T cells infected with NL4-3 (Fig. 4B and Table 2). The viral suppression ability of these primed T-cell lines was evaluated by comparing it with that of a positive-control CTL clone, 12B (GagRI8-specific), D3 (PolSI8-specific), or C5 (GagWV8-specific), which showed a very strong viral suppression ability *in vitro* (27, 34). Most of the primed T-cell lines specific for GagRI8, GagWV8, or PolSI8 epitopes exhibited high viral suppression ability, which was approximately 80% of that of the positive-control clone (Fig. 4C). Taken together, these results showed that 3′3′-cGAMP could effectively prime highly functional CD8^+^ T cells specific for HLA-B*52:01-restricted protective immunodominant epitopes from naive T cells.

**TABLE 2:** Absolute concentration of HIV-1 p24 Ags in viral replication suppression assay^*a*^

Donor	E:T ratio	Concn of HIV-1 p24 antigen (ng/ml)			
		GagRI8	PolSI8	GagWV8	Env77-85
With CTL					
U-30	1	52.0 ± 18.6	40.3 ± 9.6	39.2 ± 9.4	
	0.1	65.6 ± 12.9	52.5 ± 28.6	59.8 ± 10.3	
U-75	1	41.4 ± 0.7	62.0 ± 15.3	42.7 ± 8.0	
	0.1	54.6 ± 22.3	67.6 ± 17.2	86.3 ± 86.3	
U-62	1	10.8 ± 3.4	44.8 ± 21.3		
	0.1	14.3 ± 6.7	46.3 ± 2.7		
U-70	1	0.6 ± 0.1			
	0.1	3.8 ± 1.7			
U-52	1		16.5 ± 3.1		
	0.1		41.6 ± 18.7		
U-36	1		46.4 ± 5.0		
	0.1		55.6 ± 7.2		
Positive control	1	3.6 ± 1.8	23.0 ± 2.6	23.7 ± 1.3	
	0.1	0.9 ± 0.3	51.3 ± 5.1	33.5 ± 2.6	
Negative control	1				116.8 ± 4.8
	0.1				116.0 ± 6.6
Without CTL	0	117.4 ± 2.1	117.4 ± 2.1	117.4 ± 2.1	117.4 ± 2.1

a CD4^+^ T cells from HLA-B*52:01^+^ healthy individuals were infected with NL4-3 and then cocultured with HLA-B*52:01-restricted T-cell lines primed with cGAMP, a positive-control clone (GagRI8-, PolSI8-, or GagWV8-specific CTL clone), and a negative-control clone (HLA-B*35:01-restricted Env77-85-specific CTL clone) established from HIV-1-infected individuals at effector-to-target cell (E:T) ratios of 1:1 and 0.1:1. HIV-1 p24 Ags in the culture supernatant on day 5 postinfection were measured by conducting an enzyme immunoassay.

**FIG 5:**
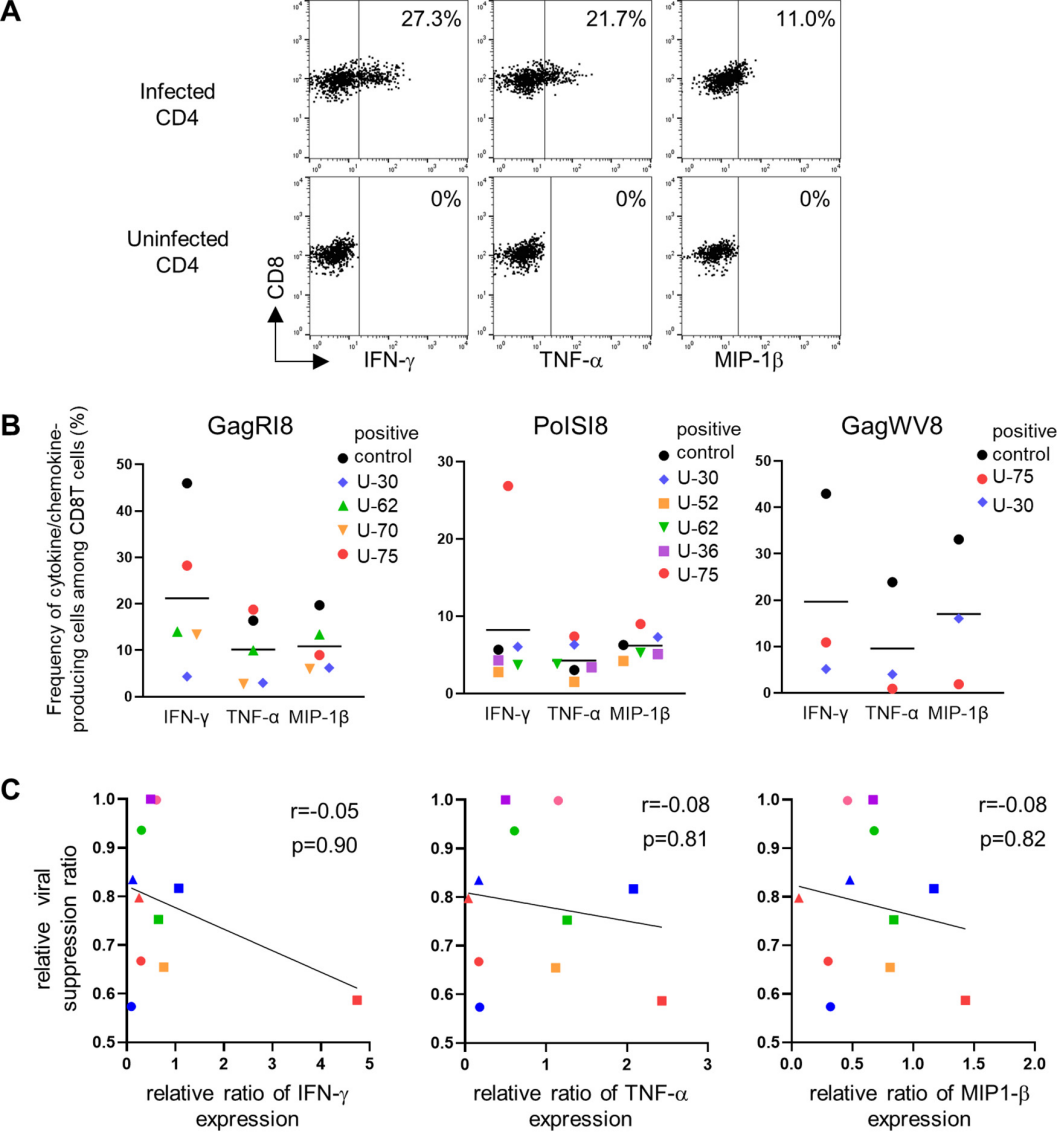
Cytokine production of HLA-B*52:01-restricted protective epitope-specific CD8^+^ T cells primed with 3′3′-cGAMP from naive T cells. The ability of 3′3′-cGAMP-primed T-cell lines specific for GagRI8, GagWV8, or PolSI8 to produce cytokines/chemokine in response to HIV-1-infected CD4^+^ T cells was evaluated. NL4-3-infected CD4^+^ T cells from an HLA-B*52:01^+^ donor were incubated for 6 h with 3′3′-cGAMP-primed T-cell lines or with a positive-control CTL clone, 12B (GagRI8 specific), D3 (PolSI8 specific), or C5 (GagWV8 specific), established from an HIV-1-infected individual. The frequency of p24 antigen-positive cells among CD4^+^ T cells infected with NL4-3 was 36.6%. (A) Representative cytokine/chemokine staining of 3′3′-cGAMP-primed T-cell line specific for GagRI8 from an HIV-1-seronegative individual (U-75). (B) Summarized results for the frequency of 3′3′-cGAMP-primed T-cell lines and control clone expressing IFN-γ, TNF-α, or MIP-1β. Experiments were performed in triplicate. Each dot represents the average (*n* = 3) frequency of cells expressing each cytokine or chemokine among the CD8^+^ T cells in each individual. Horizontal bars indicate median values. (C) Correlation between relative viral suppression ratio and relative cytokine production ratio of 3′3′-cGAMP-primed T-cell lines specific for GagRI8, GagWV8, or PolSI8. Relative cytokine production ratio was calculated as follows: frequencies of 3′3′-cGAMP-primed T-cell line expressing cytokines/those of control CTL clone expressing them. Statistical analysis was conducted by use of the Spearman correlation test.

We next investigated the ability of the primed T-cell lines specific for epitope to produce cytokines such as IFN-γ and tumor necrosis factor alpha (TNF-α), and the chemokine MIP-1β, which exert antiviral, inflammatory, and regulatory functions against HIV-1 (40–42). We used HIV-1-infected CD4^+^ T cells as stimulator cells to evaluate the ability of these T cells to recognize epitopes near the *in vivo* condition. These T cells had the ability to effectively produce these cytokines and chemokine in response to HIV-1-infected CD4^+^ T cells (Fig. 5A and B). We next analyzed the correlation between the viral suppression ability of and cytokine/chemokine production by these primed T cells. No significant correlation between these 2 abilities was found (Fig. 5C), suggesting that the ability of these primed T cells to produce these cytokines and chemokine may not have been mainly involved in their viral suppression ability.

### Production of cytolytic effector molecules in 3′3′-cGAMP-primed CD8^+^ T cells specific for protective epitopes.

**FIG 6:**
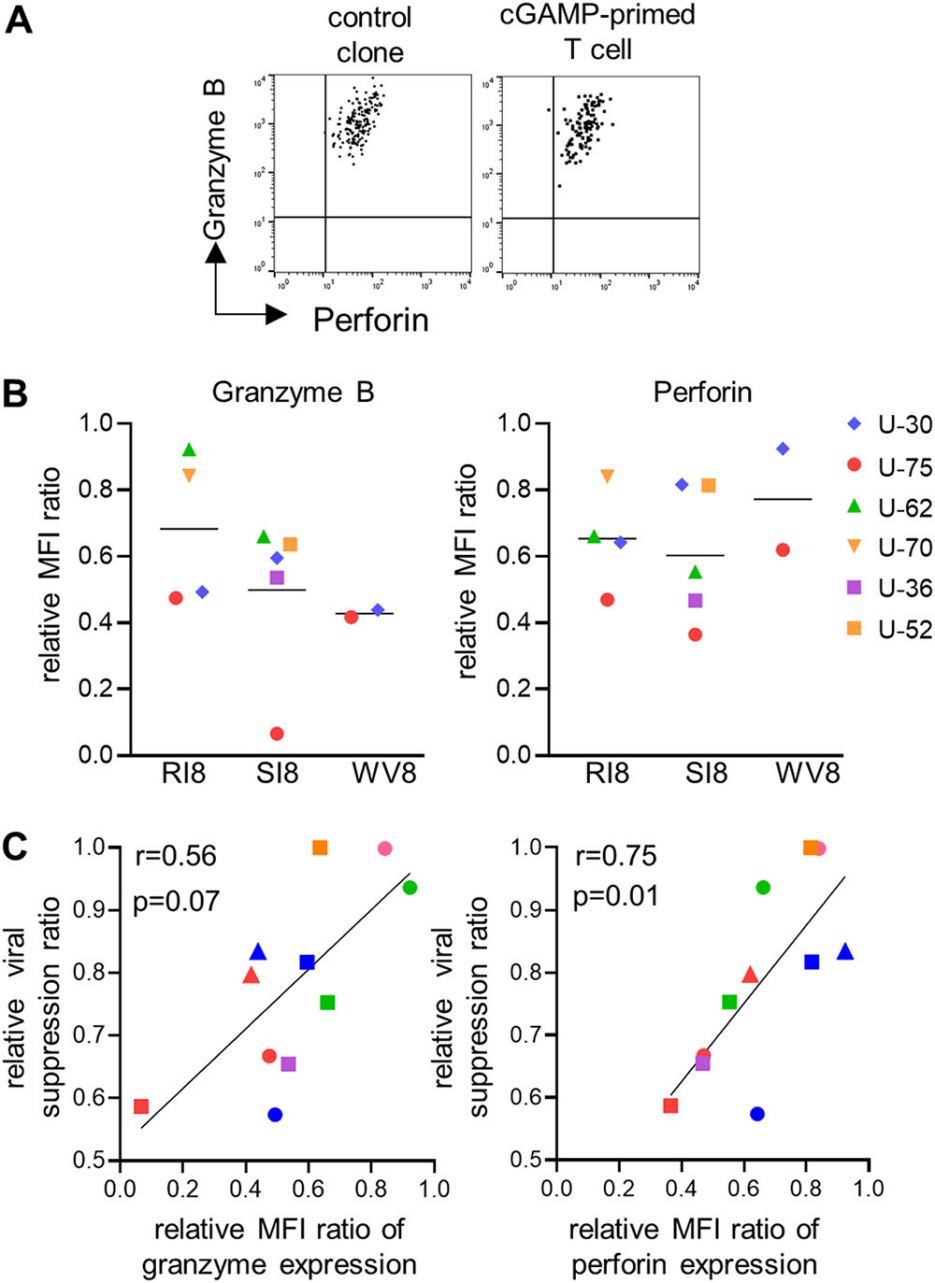
Expression levels of granzyme B and perforin in HLA-B*52:01-restricted protective epitope-specific CD8^+^ T cells primed with 3′3′-cGAMP from naive T cells. The expression levels of intracellular granzyme B and perforin in 3′3′-cGAMP-primed CD8^+^ T-cell lines specific for GagRI8, GagWV8, or PolSI8 and in a control CTL clone, 12B (GagRI8 specific), D3 (PolSI8 specific), or C5 (GagWV8 specific), without antigen stimulation were measured by performing the intracellular staining assay. (A) Representative results of staining of granzyme B and perforin in a 3′3′-cGAMP-primed T-cell line specific for GagRI8 from an HIV-1-seronegative individual (U-70) and the control clone established from an HIV-1-infected individual. (B) Evaluation of expression levels of granzyme B and perforin in 3′3′-cGAMP-primed T-cell lines in terms of relative MFI ratio. The relative MFI ratio was calculated as MFI for primed T-cell lines/that for the control clone. (C) Correlation between relative viral suppression ratio and relative expression levels of granzyme B and perforin in 3′3′-cGAMP-primed T-cell lines specific for GagRI8, GagWV8, or PolSI8. Each dot represents a T-cell line from each individual. Statistical analysis was conducted by use of the Spearman correlation test.

To clarify other potent effectors for their viral suppression ability, we further analyzed the expression levels of intracellular granzyme B and perforin in primed T-cell lines and compared them with those in a positive-control T-cell clone with strong viral suppression ability (Fig. 6A and B). T-cell lines specific for these 3 epitopes expressed granzyme B and perforin, though the levels of these molecules in the T-cell lines were relatively lower than those in the control clone. We next investigated whether the amount of intracellular perforin or granzyme B in the T-cell lines was correlated with their viral suppression ability. The viral suppression ability of the T-cell lines was significantly correlated with the expression level of perforin and showed a trend for a positive correlation with that of granzyme B (Fig. 6C). Thus, the expression of these effector molecules was correlated with the viral suppression ability of the primed T-cell lines.

**FIG 7:**
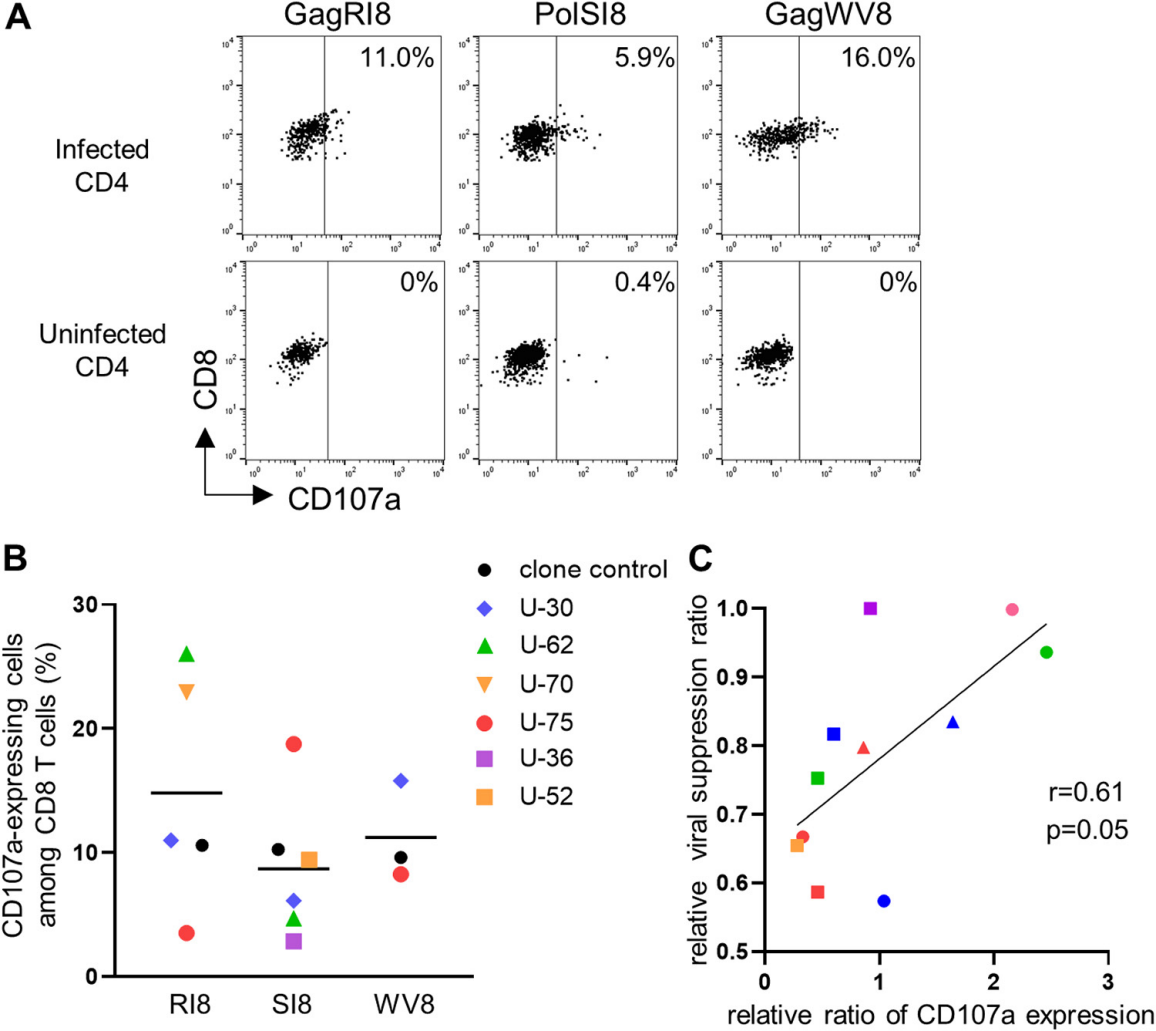
CD107a expression in HLA-B*52:01-restricted protective epitope-specific CD8^+^ T cells primed with 3′3′-cGAMP upon antigen stimulation. Expression level of CD107a in 3′3′-cGAMP-primed CD8^+^ T-cell lines specific for GagRI8, GagWV8, or PolSI8 in response to HIV-1-infected CD4^+^ T cells. NL4-3-infected CD4^+^ T cells from an HLA-B*52:01^+^ donor were incubated for 6 h with 3′3′-cGAMP-primed T-cell lines or with a control CTL clone, 12B (GagRI8 specific), D3 (PolSI8 specific), or C5 (GagWV8 specific), established from an HIV-1-infected individual. The frequency of p24 antigen-positive cells among CD4^+^ T cells infected with NL4-3 was 36.6%. The expression level of cell surface CD107a in 3′3′-cGAMP-primed T-cell lines and control CTL clones was measured by flow cytometry. (A) Representative results of staining of CD107a in a 3′3′-cGAMP-primed T-cell line from an HIV-1-seronegative individual (U-30). (B) Summarized results for the frequency of 3′3′-cGAMP-primed T-cell lines and a control clone expressing CD107a. Experiments were performed in triplicate. Each dot represents the average (*n* = 3) frequency of cells expressing CD107a among the CD8^+^ T cells in each individual. Horizontal bars indicate median values. (C) Correlation between relative viral suppression ratio and relative CD107a expression ratio in 3′3′-cGAMP-primed T-cell lines specific for GagRI8, GagWV8, or PolSI8. The relative CD107a expression was calculated as follows: frequencies of 3′3′-cGAMP-primed T-cell line expressing CD107a/those of a control CTL clone expressing it. Statistical analysis was conducted by use of the Spearman correlation test.

To verify that intracellular granzyme B and perforin could be released from primed T cells in an antigen-specific manner, we investigated the expression of the degranulation marker CD107a, which is associated with the release of lytic granule proteins (43, 44). After 6 h of stimulation with HIV-1-infected CD4^+^ T cells, CD107a expression was measured. All of epitope-specific T-cell lines expressed CD107a in response to HIV-1-infected CD4^+^ T cells (Fig. 7A and B), indicating that these T cells had released cytotoxic effector molecules such as perforin and granzyme B immediately upon antigen-specific stimulation. The expression level of CD107a showed a trend for a positive correlation with the viral suppression ability of the cells (Fig. 7C). These results together suggest that these T cells primed with 3′3′-cGAMP could suppress HIV-1 replication mainly via lytic granule-mediated killing.

## DISCUSSION

Our previous study demonstrated that the STING ligand 3′3′-cGAMP enables priming of HLA-A*24:02-restricted HIV-1 NefRF10-specific CD8^+^ T cells with a strong effector function from naive T cells via STING ligand-mediated production of type I IFN (24). This study suggested that 3′3′-cGAMP acts as an adjuvant to induce more potent T-cell immunity against HIV-1. However, it remained unclear whether this ligand could induce highly functional CD8^+^ T cells specific for other HIV-1 epitopes from naive T cells, especially those protective ones. As T cells specific for HIV-1-protective epitopes have a strong ability to suppress HIV-1 replication, priming of these T cells from naive T cells would be expected to serve as effector cells in a cure treatment such as a shock-and-kill therapy. In the present study, we therefore investigated the 3′3′-cGAMP-mediated priming of CD8^+^ T cells specific for 4 HLA-B*52:01 protective epitopes as well as those for 2 protective epitopes restricted by HLA-C*12:02 and showed that CD8^+^ T cells specific for 3 HLA-B*52:01-restricted epitopes were primed from naive T cells in HIV-1-seronegative individuals carrying the HLA-B*52:01-C*12:02 haplotype. Since all of HLA-B*52:01-restricted CD8^+^ T cells specific for these 3 protective epitopes exhibited a strong ability to suppress HIV-1 replication, they would be expected to be elicited in individuals with this haplotype if they were immunized with this epitope antigen together with cGAMP.

A previous study on CD8^+^ T cells specific for 3 hepatitis C virus (HCV) epitopes showed that the frequency of naive precursor T cells specific for the HCV epitopes is related to that of the specific T cells in chronically HCV-infected patients (45). We therefore speculated that the efficacy of HIV-1-specific T-cell induction from naive T cells would be associated with the immunodominance (breadth and magnitude) of HIV-1-specific T cells in HIV-1-infected individuals. Our previous study showed that T-cell responses to GagRI8, GagMI8, GagWV8, and PolSI8 were detected at 60% (60/100), 42% (42/100), 43% (43/100), and 43% (43/100), respectively, in HLA-B*52:01^+^ individuals chronically infected with HIV-1 subtype B (26). However, CD8^+^ T cells specific for the MI8 epitope were not induced from naive T cells in any of the HIV-1-seronegative individuals in the present study. The binding affinity of the MI8 peptide for the HLA-B*52:01 molecule was much lower than that of the other 3 peptides. This result suggested that the failure of induction of MI8-specific T cells from naive T cells resulted from lower presentation of this epitope by DCs. Since MI8-specific CD8^+^ T cells were efficiently elicited in chronically HIV-1-infected individuals, the binding ability of MI8 peptide to HLA-B*52:01 may not critically affect the efficacy of T-cell induction in a chronic infection. A previous study showed that the magnitude of the primary T-cell response is influenced by the amount and duration of presentation of the relevant peptide-HLA ligands detected by antigen-presenting cells (46–49). Since a continuous high viral load is seen during chronic HIV-1 infection, persistent exposure to HIV-1 antigens may compensate for the small amount of antigen presentation due to weak binding of MI8 epitope peptide to HLA-B*52:01. Several studies showed that the immunodominance of HIV-1-specific CD8^+^ T-cell responses in a chronic infection is different from that in an acute one (50, 51), suggesting that duration of antigen exposure may be involved in this difference.

A previous study showed that T-cell responses to PolIY11 and NefMY9 are detectable in 25% of HLA-C*12:02^+^ individuals chronically infected with HIV-1 subtype B but that those to EnvRL9 are found in 65% of these individuals (36). Since EnvRL9-specific T cells were elicited more frequently than those specific for PolIY11 and NefMY9, we also analyzed the priming of EnvRL9-specific T cells. However, CD8^+^ T cells specific for all 3 HLA-C*12:02-restricted epitopes were not primed from any of the HIV-1-seronegative individuals tested, even though those specific for HLA-B*52:01-restricted epitopes were successfully primed from the same individuals in 6 cases. Since cell surface expression levels of HLA-C are lower than those of HLA-B and HLA-A on uninfected cells and even on cells infected with HIV-1 (37–39), antigen presentation by HLA-C molecules could be weaker than that by HLA-A or HLA-B ones. We therefore speculated that it would be difficult to prime CD8^+^ T cells specific for HLA-C-restricted epitopes from naive T cells due to lower presentation by the HLA-C allele. Indeed, we confirmed that the expression of HLA-C*12:02 on DCs and T cells from an HIV-1-seronegative individual was lower than that of HLA-B*52:01 on these cells.

On the other hand, persistent antigen exposure may enable the induction of HLA-C-restricted CD8^+^ T cells in HIV-1-infected individuals as discussed above. The expression level of HLA-C alleles is positively correlated with the frequency of HLA-C-restricted T-cell responses in Europeans and African-Americans (52), suggesting that higher expression of HLA-C molecules results in a more efficient primary response of the HLA-C-restricted T cells in HIV-1-infected individuals. The expression level of HLA-C*12:02 remains unknown, but it might be the same as that of HLA-C*12:03, which is expressed at a medium level among HLA-C alleles (52). It can be expected that the STING ligand may prime HIV-1-specific CD8^+^ T cells restricted by other HLA-C alleles such as HLA-C C*01:02 or HLA-C C*14:02, which are highly expressed on the cell surface (52).

In the majority of HIV-1-infected individuals treated with long-term cART, HIV-1-specific effector CD8^+^ T cells are lost due to weak antigen stimulation under very low viral loads (10, 11). In addition, the remaining HIV-1-specific memory CD8^+^ T cells have ineffective or dysfunctional antiviral functions (13, 14). Therefore, it would be expected that the induction of HIV-1-specific CD8^+^ T cells with high function from naive T cells would be required for eliminating HIV-1-infected cells in individuals on cART. cGAMP-primed HLA-B*52:01-restricted CD8^+^ T cells specific for 3 epitopes showed high expression levels of perforin and had the ability to suppress HIV-1 replication. These findings allow us to expect that these HLA-B*52:01-restricted primed T cells might contribute to the elimination of the latency-reversing agent-reactivated HIV-1 reservoir in HIV-1-infected individuals on cART. We previously identified other HLA-restricted protective T-cell epitopes specific for conserved Gag and Pol regions (26–30). cGAMP-primed CD8^+^ T cells specific for these epitopes together with those for the 3 HLA-B*52:01 epitopes described here may be useful as effector T cells for eradication of latent HIV-1-infected cells in a broad number of HIV-1-infected individuals.

A previous study showed that HIV-1-specific memory CD8^+^ T cells are detectable during cART but exhibit lower cytolytic function, even HLA-B*57 or HLA-B*27 protective allele-restricted T cells (13, 14). Therefore, it is important to induce functional HIV-1-specific T cells from naive T cells in individuals under cART. In the present study, we demonstrated that HLA-B*52:01-restricted protective epitope-specific CD8^+^ T cells with strong effector functions were primed with STING ligand 3′3′-cGAMP from naive T cells, suggesting that these primed T cells may contribute to HIV-1 prevention and cure. The present study highlighted the priming with STING ligand of functional CD8^+^ T cells specific for protective epitopes and suggested that the priming of HIV-1-protective epitope-specific T cells would contribute to a shock-and-kill therapy. Priming of HIV-1-specific T cells from naive T cells in individuals with cART is important for shock-and-kill therapy, since the naive T-cell repertoire is smaller in HIV-1-infected individuals than that in uninfected ones (53). A study to recover the function of HIV-1-specific memory and effector T cells would also be important for this therapy. Thus, we expect to study the effect of STING ligand on HIV-1-specific memory and effector T cells in the near future.

## MATERIALS AND METHODS

### Subjects.

Eight HIV-1-seronegative individuals having the HLA-B*52:01-C*12:02 haplotype and 7 ones having HLA-A*24:02 were recruited for this study. This study was approved by the Ethical Committee of Kumamoto University, Japan. Written informed consent was obtained from all subjects for the collection of blood and their subsequent analysis according to the Declaration of Helsinki. Peripheral blood mononuclear cells (PBMCs) were separated from whole blood by the use of Ficoll-Paque Plus. HLA genotypes of all individuals were determined by the Luminex microbead method at the HLA laboratory (Japan).

### Cell lines.

C1R cells expressing HLA-B*52:01 (C1R-B*52:01), 721.221 cells expressing HLA-B*52:01 (721.221-B*52:01) or HLA-C*12:02 (721.221-C*12:02), and TAP2-deficient RMA-S cells expressing HLA-B*52:01 (RMA-S-B*52:01) were previously generated (33, 54, 55). These cells were cultured in RPMI 1640 medium (Thermo Fisher) containing 5% fetal calf serum (FCS) and 0.15 mg/ml of hygromycin B.

### *In vitro* priming of HIV-1-specific CD8^+^ T cells from naive T cells.

Naive CD8^+^ T-cell precursors specific for HLA-B*52:01-restricted, HLA-A*24:02-restricted, or HLA-C*12:02-restricted ones were primed *in vitro* by using the accelerated dendritic cell coculture protocol (23, 24, 56, 57). On day 0, frozen-thawed PBMCs of HIV-1-seronegative individuals having the HLA-B*52:01-C*12:02 haplotype or HLA-A*24:02 were suspended at 5 × 10^6^ cells/well in 24-well tissue culture plates containing AIM-V medium (Invitrogen) supplemented with Flt3L (50 ng/ml; R&D Systems). AIM-V medium supplemented with a 10 μM concentration of each peptide and STING ligand 3′3′-cGAMP (10 μg/ml; InvivoGen) was added on day 1, and then FCS was further added to 10% by volume per well on day 2. On day 10, the cells were collected and then stained with epitope-specific tetramers.

### Tetramer staining.

HLA-B*52:01-RI8, -MI8, -SI8, or -WV8 peptide tetrameric complexes (tetramers) and HLA-A*24:02-RF10, HLA-C*12:02-IY11, -MY9, or -RL9 complexes were generated as previously described (58). CD8^+^ T cells specific for a given epitope were stained with phycoerythrin (PE)-conjugated epitope-specific tetramers at 37°C for 30 min. The cells were then washed twice with RPMI 1640 medium containing 5% FCS (R5), followed by staining with allophycocyanin (APC)-conjugated anti-CD8 MAb (Dako, Denmark) and 7-aminoactinomycin D (7-AAD; BD Pharmingen) at 4°C for 30 min. Finally, the cells were washed twice with R5 and then analyzed by using a FACS Canto II. Primed T cells unstained with tetramer were used as a negative control to determine gating of the tetramer^+^ population. The tetramer^+^ population was determined based on the gating of the negative control (<0% of tetramer^+^ cells).

### Establishment of CD8^+^ T-cell lines specific for epitopes.

CD8^+^ T cells specific for GagRI8, PolSI8, or GagWV8 primed from HIV-1-seronegative individuals were stained with PE-conjugated epitope-specific tetramers, APC-conjugated anti-CD8 MAb, and 7-AAD. The population of tetramer^+^ CD8^+^ 7-AAD^−^ cells was sorted in U-bottomed 96-well microtiter plates by using a FACS Aria (BD Biosciences). Each well contained 200 μl of cell mixture (1 × 10^5^ irradiated allogeneic PBMCs from healthy donors and 1 × 10^5^ irradiated C1R-B*52:01 cells prepulsed with the peptide at 100 nM, 20 ng/ml of human recombinant interleukin 2 (rIL-2; ProSpec), and 2.5% phytohemagglutinin soup). CTL clones specific for HLA-B*52:01-restricted GagRI8, GagWV8, or PolSI8 were established from HIV-1-infected individuals as previously described (27, 34).

### HLA stabilization assay.

The ability of peptide to bind to HLA-B*52:01 was measured by using RMA-S-B*52:01 cells, as previously described (55, 59). RMA-S-B*52:01 cells were incubated at 26°C for 16 h, then pulsed with various concentrations of peptides at 26°C for 1 h, and subsequently incubated at 37°C for 3 h. Thereafter, the cells were stained with anti-HLA class I α3 domain MAb TP25.99 (60) and fluorescein isothiocyanate (FITC)-conjugated sheep anti-mouse IgG (Jackson ImmunoResearch). The mean fluorescence intensity (MFI) was measured by using the FACS Canto II.

### Surface expression of HLA class I molecules on DCs and T cells.

We first investigated the concentrations of anti-B5 MAb (4D12) and anti-HLA-C MAb (DT9) that bound to the same number of HLA-B*52:01 and HLA-C*12:02 molecules, respectively. HLA class I-deficient 721.221 cells transfected with HLA-B*52:01 or HLA-C*12:02 gene (721.221-B*52:01 or 721.221-C*12:02 cells) were used to evaluate these concentrations. These cells were stained with various concentrations of 4D12, DT9, and TP25.99 anti-HLA class I α3 domain MAb followed by PE-conjugated anti-mouse IgG (BioLegend). The MFI of 721.221-HLA-B*52:01 and 721.221-HLA-C*12:02 cells stained with these antibodies was measured by flow cytometry, and then the relative MFI was calculated as follows: MFI of cells stained with MAb/MFI of cells unstained with MAb. To quantify binding affinity of 4D12 (61) and DT9 (39), we normalized the relative MFI of cells stained with 4D12 and DT9 to that of cells stained with TP25.99 by calculating relative binding ratio as follows: the relative MFI of 4D12 or DT9 MAb/the relative MFI of TP25.99 MAb. The concentration of 4D12 and DT9 providing the same relative binding ratio was used to evaluate the surface expression of HLA-B*52:01 or HLA-C*12:02 on the cells.

PBMCs isolated from 3 individuals who were homozygous for the HLA-B*52:01-C*12:02 haplotype were stimulated with Flt3 ligand for 24 h, followed by stimulation with 3′3′-cGAMP for 24 h or 48 h. The cells stimulated with 3′3′-cGAMP for 0 h, 24 h or 48 h were collected and then stained with diluted anti-B5 MAb 4D12, anti-HLA-C MAb DT9, or isotype control followed by PE-conjugated anti-mouse IgG (BioLegend). These cells were stained with the reagents of a LIVE/DEAD fixable near-infrared (IR) dead cell stain kit (Invitrogen) and FITC-conjugated lineage cocktail 1 (Lin1, CD3/CD14/CD16/CD19/CD20/CD56; BD Bioscience), peridinin chlorophyll protein (PerCP)-conjugated HLA-DR MAb (BioLegend), PE-Cy7-conjugated CD123 MAb (BioLegend), APC-conjugated CD11c MAb (BioLegend) for DCs (62), or FITC-conjugated CD3 MAb for T cells. The MFI was measured by using the FACS Canto II. Gated PBMCs negative for expression of Lin markers and positive for expression of HLA-DR were analyzed for CD11c and CD123. HLA-DR^+^ Lin^−^ populations were divided into different subsets: CD11c^+^ CD123^lo^ (myeloid DCs [mDCs]) and CD11c^−^ CD123^hi^ (plasmacytoid DCs [pDCs]).

### HIV-1 replication suppression assay.

The suppression ability of epitope-specific CD8^+^ T-cell lines or CTL clones was measured as previously described (63, 64). CD4^+^ T cells isolated from PBMCs obtained from an HLA-B*52:01^+^ healthy donor were incubated with HIV-1 strain NL4-3. After 5 h, the infected CD4^+^ T cells were cocultured with epitope-specific CD8^+^ T-cell lines or CTL clones at effector-to-target cell (E:T) ratios of 1:1, 0.1:1, and 0:1. On day 3 to day 6 postinfection, the concentration of p24 antigen in the collected culture supernatant was measured by use of an enzyme-linked immunosorbent assay (ELISA) kit (HIV-1 p24 antigen [Ag] ELISA kit; ZeptoMetrix). The percent inhibition of HIV-1 replication was calculated as follows: percent suppression = (1 − concentration of p24 Ag in the supernatant of HIV-1-infected CD4^+^ T cells cultured with epitope-specific CD8^+^ T cells/concentration of p24 Ag in the supernatant of HIV-1-infected CD4^+^ T cells cultured without the T cells) × 100.

### Intracellular cytokine staining assay.

HLA-B*52:01-restricted CD8^+^ T-cell lines or clones were stimulated with NL4-3-infected CD4^+^ T cells from an HLA-B*52:01^+^ healthy donor at an effector-to-stimulator ratio of 1:1 for 6 h at 37°C. APC-conjugated anti-CD107a MAb (BioLegend) and brefeldin A (10 μg/ml; Sigma-Aldrich) were added 1 h and 2 h after stimulation, respectively. After a 6-h incubation, the cells were stained with 7-AAD and FITC-conjugated anti-CD8 MAb (Dako) at 4°C for 30 min, fixed with 4% paraformaldehyde solution at 4°C for 20 min, and then made permeable with 0.1% saponin buffer by incubation at 4°C for 10 min. Thereafter, these cells were stained with PE-conjugated anti-IFN-γ MAb (BioLegend), PE-Cy7-conjugated TNF-α MAbs (BD Pharmingen), and APC-H7-conjugated MIP-1β MAbs (BD Pharmingen) at room temperature for 30 min. Nonspecific production of cytokines was excluded by subtracting the data of the negative control, which was the same sample stimulated with uninfected CD4^+^ T cells and stained with the same MAbs.

### Intracellular staining of perforin and granzyme B.

Epitope-specific CD8^+^ T-cell lines or CTL clones stained with PE-conjugated epitope-specific tetramer, PE-Cy7-conjugated CD8 MAb, and 7-AAD were fixed with 4% paraformaldehyde solution and then rendered permeable with 0.1% saponin buffer. Thereafter, the cells were stained with Alexa Fluor 647-labeled anti-Granzyme B (BD Pharmingen) and Alexa Fluor 488-labeled anti-perforin (BD Pharmingen). All stained cells were analyzed by using the FACS Canto II.

### Statistics.

Statistical analyses were performed by using Prism software (GraphPad). Groups were compared by using the paired *t* test or unpaired *t* test. Correlations were determined with the Spearman rank test. *P* values of <0.05 were considered significant.
